# Dosage concentration and pulsing frequency affect the degradation efficiency in simulated bacterial polycyclic aromatic hydrocarbon-degrading cultures

**DOI:** 10.1007/s11356-023-26546-9

**Published:** 2023-04-04

**Authors:** Anjela L. Vogel, Katharine J. Thompson, Sara Kleindienst, Christiane Zarfl

**Affiliations:** 1 Department of Geosciences, Eberhard Karls University of Tübingen, Schnarrenbergstr. 94-96, 72076 Tübingen, Germany; 2 Department of Environmental Microbiology, Institute for Sanitary Engineering, Water Quality and Solid Waste Management (ISWA), University of Stuttgart, Stuttgart, Germany

**Keywords:** Marine oil pollution, Chronic low-concentration pollution, Biodegradation, Chemical fate modelling, Bacterial growth model, Simulation of batch experiments

## Abstract

A major source of anthropogenic polycyclic aromatic hydrocarbon (PAH) inputs into marine environments are diffuse emissions which result in low PAH concentrations in the ocean water, posing a potential threat for the affected ecosystems. However, the remediation of low-dosage PAH contaminations through microbial processes remains largely unknown. Here, we developed a process-based numerical model to simulate batch cultures receiving repeated low-dosage naphthalene pulses compared to the conventionally used one-time high-dosage. Pulsing frequency as well as dosage concentration had a large impact on the degradation efficiency. After 10 days, 99.7%, 97.2%, 86.6%, or 83.5% of the 145 mg L^−1^ naphthalene was degraded when given as a one-time high-dosage or in 2, 5, or 10 repeated low-concentration dosages equally spaced throughout the experiment, respectively. If the simulation was altered, giving the system that received 10 pulses time to recover to 99.7%, pulsing patterns affected the degradation of naphthalene. When pulsing 10 days at once per day, naphthalene accumulated following each pulse and if the degradation was allowed to continue until the recovered state was reached, the incubation time was prolonged to 17 days with a generation time of 3.81 days. If a full recovery was conditional before the next pulse was added, the scenario elongated to 55 days and generation time increased to 14.15 days. This indicates that dissolution kinetics dominate biodegradation kinetics, and the biomass concentration of PAH-degrading bacteria alone is not a sufficient indicator for quantifying active biodegradation. Applying those findings to the environment, a one-time input of a high dosage is potentially degraded faster than repeated low-dosage PAH pollution and repeated low-dosage input could lead to PAH accumulation in vulnerable pristine environments. Further research on the overlooked field of chronic low-dosage PAH contamination is necessary.

**Supplementary Information:**

The online version contains supplementary material available at 10.1007/s11356-023-26546-9.

## Introduction

Approximately 1.3 million tons of hydrocarbons are emitted through anthropogenic and natural sources to marine environments every year (National Research Council, 2003). Most prominently known among anthropogenic sources are accidental oil spills and disasters like the Deepwater Horizon accident in 2010, which generate high public and scientific attention. Consequently, current research mainly focuses on one-time high-hydrocarbon input scenarios, however, these account for only 9% of the total emitted hydrocarbons. Another research focus is often natural oil seeps which are continuously polluted extreme environments at the seafloor, comprising 48% of global hydrocarbon emissions (National Research Council, 2003). In contrast, anthropogenic, repeating, diffuse and often low-dosage hydrocarbon emissions account for up to 40% of the total but are underrepresented in research. This is mainly because their location and timing are highly variable and they occur in pulses, e.g., due to ships passing on a shipping route, rain events causing increased run-off or atmospheric deposition from burning organic matter or fuels (National Research Council 2003; Duran and Cravo-Laureau 2016). These factors make them hard to identify, quantify, and investigate in situ; thus, knowledge on the impact of repeated low-dosage hydrocarbon pollution remains scarce.

Microbial hydrocarbon degradation is a crucial process for contaminant removal from (marine) environments, such as the ocean water column (González-Gaya et al. 2019). This is well studied for high-pollution events under both oxic and anoxic conditions in aquatic systems, considering different salinities, and temperatures (Gutierrez et al. 2013; Joye et al. 2016), but is not well studied for repeated low-dosage scenarios due to the difficulties mentioned above. When studying hydrocarbons, it becomes apparent that there is a difference between aliphatic and polyaromatic hydrocarbons (PAHs). While aliphatic, chain-like hydrocarbons are degraded faster, PAHs are known to be more persistent and, due to relatively low hydrophilic properties, tend to concentrate and accumulate in organic material and organisms (Baussant et al. 2001; Landrum et al. 2003). Indeed, some PAH compounds have been listed as “Priority Pollutants” by the US EPA since the 1990s due to their persistence, acute toxicity, and carcinogenicity (Environmental Protection Agency 1993).

PAH degradation by marine bacteria is a highly relevant and well-known process, helping the environment to recover from one-time high-input hydrocarbon pollution events (Joye et al. 2016; Karthikeyan et al. 2020; Bacosa et al. 2022). Observed PAH degradation half-lives range from days to months and strongly depend on the complexity of substrate (single compound vs. mixture of hydrocarbons), temperature, UV light and composition of the microbial community. For example, Liu et al. (2016) found temperature-dependent degradation rate constants between 0.101 and 0.399 d^−1^ with PAH residuals between 34 and 75% after 50 days by performing in situ-like microcosm experiments using seawater contaminated with 200 mg L^−1^ crude oil. In comparison, a pure culture of *Cycloclasticus* spp. degraded up to 99% of 200 mg L^−1^ within 10 days under optimized conditions in a laboratory based study by Cui et al. (2014). Knowledge on the degradation dynamics of repeated low-dosage PAH contamination, however, is scarce since small changes in low PAH concentrations are more difficult to study in the laboratory due to technical limitations and biological variability that masks actual changes in low concentration.

To date, only a few studies have investigated repeated low-dosage hydrocarbon degradation in the water column of the ocean. For example, Bacosa et al. (2021) incubated seawater microcosms with different initial oil concentrations for 50 days and environmentally exposed flow-through incubators were studied with two different concentrations of weathered diesel (Ryther et al. 2021). No further in situ, in situ-like, lab-based or simulation-based studies are available so far to predict the fate of PAHs in a chronic pollution scenario. It also remains unclear what role the frequency of input and the time interval between regularly occurring pollution events play in the recovery of the system. This knowledge would be highly relevant for risk assessment and contamination management, given that many low-dosage PAH inputs are recurring pulse-like pollution events. Research on other contaminants like herbicides suggests that frequency and dosage concentration play an important role for the degradation efficiency of contaminants (Baelum et al. 2008; Lancaster et al. 2010). Nonetheless, no data has been published so far on pulsing experiments or field studies when it comes to marine hydrocarbons, or more specifically PAH degradation.

Modelling microbial PAH degradation could help start closing the knowledge gap on repeated marine low-dosage hydrocarbon pollution of the water column. Although field and laboratory-based studies are not replaceable in order to understand environmentally relevant processes, they have limits when it comes to temporal and spatial resolution, as well as number of experiments.

Therefore, conceptual models that represent our current understanding of interacting processes and driving parameters related to microbial PAH degradation in aqueous environments can be used as an alternative, ultimately, translating our experimental knowledge into mathematical equations (Soulas and Lagacherie 2001; Brimo et al. 2016). Numerical simulations can complement microbiological experimental research and help to analyse available data and derive parameter values and degradation kinetics. Based on scenario analysis, study results can be transferred to different conditions, habitats, or environmental scales and the experimental design for follow-up studies can be improved. Additionally, using mathematical model simulations with data from previously conducted experiments can help to understand dynamics in between sampling points and beyond the sampling time. This also allows the sampling design for future experiments to be adapted (if required), e.g., by choosing sampling times and scales according to the most “critical” dynamics as projected by the model. Furthermore, model simulations can be used as a first step to investigate different hypotheses, e.g., on driving parameters, to refine experimental design and generate preliminary data sets. More specifically, utilizing data on microbial degradation in one-time high-dosage scenarios (Wang et al. 2018) can inform simulations of repeated low-dosage microbial degradation. This can ultimately help to anticipate the fate of unexplored repeated low-dosage oil input within marine environments.

The overarching aim of this study was to simulate and understand recovery times of a batch culture for different PAH input scenarios, by comparing one-time high-dosage to repeated low-dosage inputs. Therefore, i) a numerical model simulating a single-strain batch culture of a known marine PAH-degrader was developed; ii) parameter values were derived by applying our model to available literature data on batch culture studies; and iii) scenarios were run to predict the dynamics of biomass growth and PAH concentrations over the course of simulated one-time high-dosage vs. repeated low-dosage degradation experiments.

## Methods

### Experimental data

Wang et al. (2018) performed one-time high-input batch culture studies with different PAHs in order to analyse the degradation pathway, on a genetic level, using the isolated marine model organism *Cycloclasticus* sp. strain P1, a strain from a species of well-known key players in marine PAH degradation (Dyksterhouse et al. 1995; Geiselbrecht et al. 1998; Dombrowski et al. 2016). They followed cell density and total PAH residual percent of the initial concentration for ten days (Fig. 1 in Wang et al. (2018)). Their growth experiment is briefly summarized here since its data are the basis for our model*; Cycloclasticus* sp. strain P1 was grown in 100 ml oxic artificial seawater medium at 28 °C on a rotary shaker (150 rpm) with 500 ppm naphthalene as the sole carbon source for 10 days. Naphthalene residuals were measured via HPLC, bacterial growth was monitored with optical density measurements at a wavelength of 600 nm (OD_600_). Further details can be found in Wang et al. (2018). We used the published growth observations as biomass concentration (after conversion from OD_600_ in cells L^−1^, Eq. ES1, SI) and naphthalene concentrations in mg L^−1^, calculated from the naphthalene residual data as input for model calibration (Table S1).

**Fig. 1:**
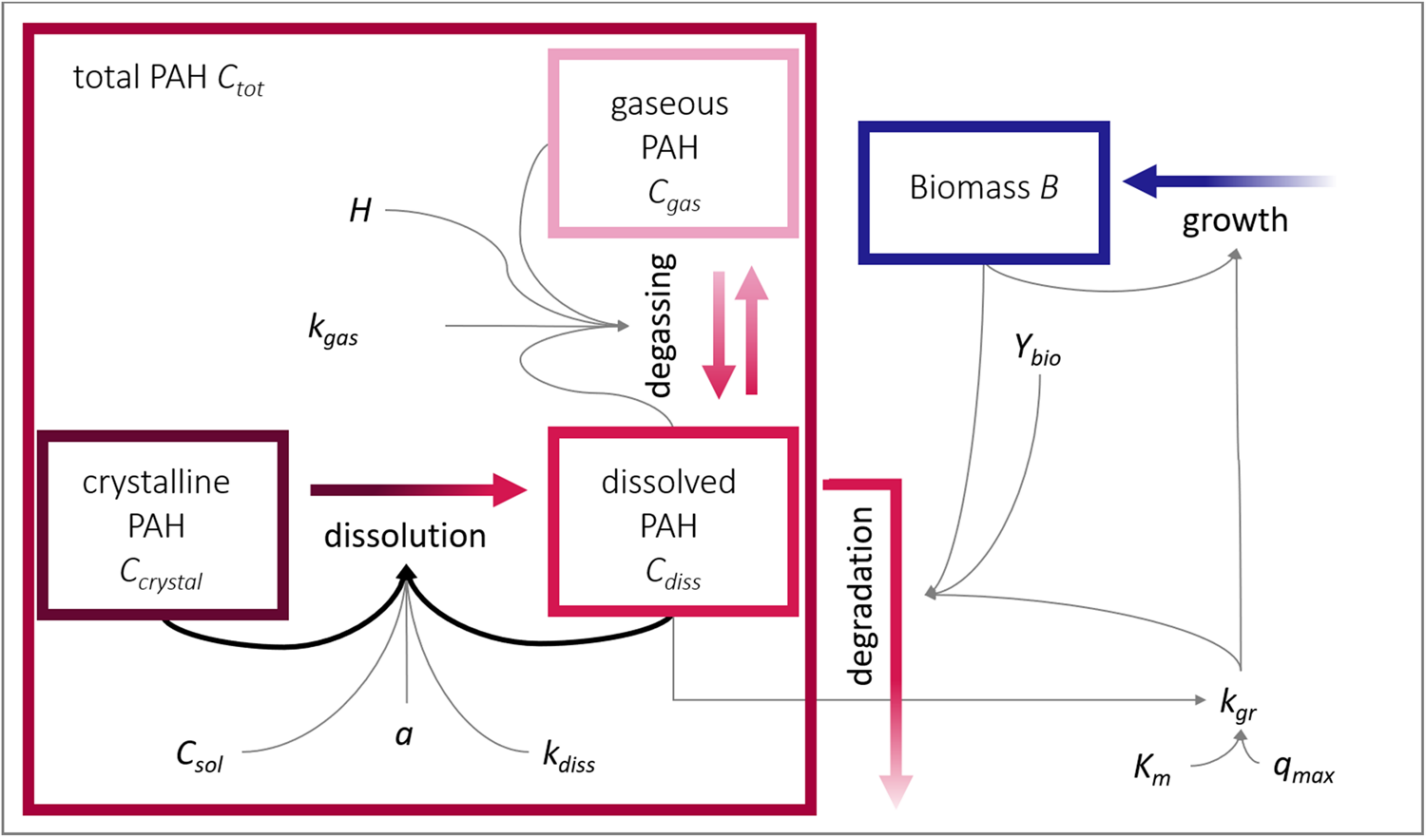
Overview of the dosage-schemes in different simulated scenarios of the reference model; number of dosages, dosage interval and dosage concentration for scenarios S1 to S6

### Conceptual model

PAH is assumed to be present in different states, i.e., gaseous, dissolved in the aqueous phase, and crystalline (Fig. 1). In the model, these PAH fractions, as concentrations (mg L^−1^), were assigned to three of the state variables of the model, *C*_*gas*_, *C*_*crystal*_, and *C*_*diss*_, respectively. After being emitted as a pure compound or in a mixture with different PAHs and solvents, evaporation and spreading/dilution of the crude oil compounds would lead to a decrease in the PAH concentration under environmental conditions. Aside from photooxidation at the seawater surface, biodegradation is the main process that removes PAHs from the water column. Therefore, the fourth state variable of the model is assigned to the biomass of the PAH-degrading bacteria, *B* in cells L^−1^.

The model is designed to represent batch experimental conditions and is based on model concepts developed by Monod (1949), Volkering et al. (1992), Grimberg et al. (1996), Knightes and Peters (2003), and Brimo et al. (2016). When simulating environmental processes in laboratory batch cultures incubated in the dark, photooxidation as well as spreading and dilution of PAHs can be neglected. The following processes, that link the four state variables, are considered in the initial model structure, that from here on is called reference model (M_ref_):

PAH dissolution: Dissolution of the crystalline PAH (*C*_*crystal*_) into dissolved PAH (*C*_*diss*_) is driven by the gradient between the aqueous saturation concentration, i.e., the solubility, of the PAH (*C*_*sol*_) and the dissolved concentration (*C*_*diss*_). The rate of mass transfer across the solid–liquid interface depends, in addition, on the specific surface area of the PAH crystals *a* (surface area per unit volume, in cm^−1^) and the mass transfer coefficient for dissolution *k*_*diss*_ (in cm h^−1^) which is, in fact, a function of chemical and hydrodynamic properties. Since a batch culture is a closed system with limited available PAH, *C*_*crystal*_ needs to be accounted for as a limited resource for dissolution while *C*_*sol*_ is considered as the capacity-term for maximum dissolution of PAH.

PAH degassing: Unitless Henry coefficients of volatile PAHs range from 8.88·﻿10^–6^ to 3.1110^–2^ (naphthalene to picene, (Sander 2015)) and indicate a distribution process from the aqueous to the gas phase. It remains to be determined if volatilization of PAHs into the headspace is a relevant process in a closed batch system under laboratory conditions. Thus, degassing is considered in the reference model structure and follows the concentration gradient between the dissolved (*C*_*diss*_) and the gaseous PAH (*C*_*gas*_).

PAH degradation: Aerobic PAH-degrading bacteria can oxidize hydrocarbons via complex metabolic pathways, thereby using them as carbon and/or energy source for biomass production and dissimilatory processes which result in CO_2_ production (Bouchez et al. 1996; Baboshin and Golovleva 2012; Dombrowski et al. 2016). Considering the lipophilic properties of PAHs and high ionic strength of seawater, solubility is limited, making it a potential rate limiting step in microbial degradation since uptake into the cell and, therefore, bioavailability is strongly linked to the dissolution of compounds. Thus, only the dissolved fraction of PAHs is considered to undergo degradation. PAH degradation is linked to biomass growth and follows Monod kinetics assuming a constant biomass yield per PAH mass (*Y*_*bio*_ in mg_BIO_ mg_PAH_^−1^).

Bacterial growth: Growth of bacterial biomass *B* (cells L^−1^) is assumed to follow Monod kinetics with the Monod constant (or half-saturation constant) *K*_*m*_ (mg L^−1^) and maximum PAH degradation rate *q*_*max*_ in mg_PAH_ mg_BIO_^−1^ h^−1^. As described above, the biomass yield *Y*_*bio*_ is assumed to be constant. Growth is considered to be net growth, i.e., explicit cell death does not play a role during the simulated 10-day period. Production of emulsifying compounds by bacteria is neglected in our model, as is sorption of PAH to biomass.

### Model equations

The mass balance model, as outlined above, was formalized into the following system of four differential equations that simulate the dynamics of microbial biomass *B* (cells L^−1^) and hydrocarbon concentration in aqueous (*C*_*dis*s_ in mg L^−1^), crystalline (*C*_*crystal*_, in mg L^−1^) and gaseous (*C*_*gas*_, in mg L^−1^) states in a PAH degrading, marine batch culture.

1 $$\frac{dB}{dt}={k}_{gr}\bullet B$$

2 $$\begin{aligned} \frac{d{C}_{diss}}{dt}={k}_{aq}\bullet a\bullet {C}_{crystal}\bullet \left(1-\frac{{C}_{diss}}{{C}_{sol}}\right)\\ -\frac{{k}_{gr}}{{Y}_{bio}} \bullet \left(B \bullet {10}^{-9}\right)-{k}_{gas}\bullet \left({C}_{diss}\bullet H-{C}_{gas}\right) \end{aligned}$$

3 $$\frac{d{C}_{crystal}}{dt}=-{k}_{diss}\bullet a\bullet {C}_{crystal}\bullet \left(1-\frac{{C}_{diss}}{{C}_{sol}}\right)$$

4 $$\frac{d{C}_{gas}}{dt}={k}_{gas}\bullet \left({C}_{diss}\bullet H-{C}_{gas}\right)$$

with the growth rate constant *k*_*gr*_ (h^−1^) as a function of the dissolved PAH concentration *C*_*diss*_ following Monod kinetics

5 $${k}_{gr}={ q}_{max} \bullet \left(\frac{{C}_{diss}}{{K}_{m}+{C}_{diss}}\right)$$

and the mass transfer coefficient for dissolution *k*_*diss*_ (cm h^−1^), the specific surface area *a* (cm^−1^) of the crystalline PAH, the solubility, i.e., saturation concentration of the PAH in the aqueous phase *C*_*sol*_ (mg L^−1^), the biomass yield *Y*_*bio*_ (mg_BIO_ mg_PAH_^−1^), the mass transfer coefficient for volatilization *k*_*gas*_ (h^−1^), the dimensionless Henry coefficient *H* (-), the maximum growth rate constant *q*_*max*_ (h^−1^) of the bacteria, and the Monod half-saturation constant *K*_*m*_ (mg L^−1^). The factor 10^–9^ in the degradation term of *C*_*diss*_ (Eq. 2) is a conversion factor for the microbial cellular biomass, i.e., biomass per cell (mg cell^−1^). This system of ordinary differential equations was defined as the reference model structure and was solved numerically with MATLAB R2018a using the in-built solver ode15s.

Model alternatives and sensitivity were analyzed by switching on and off subsets of transfer pathways (processes) between the variables of PAH states and bacterial biomass *B*, varying parameter dependencies and adopting mathematical descriptions of the assumed kinetics where reasonable.

### Model alternatives and sensitivity analysis

A model alternative was developed to investigate the structural uncertainty due to assumptions made about processes and their kinetics. The model alternative maintains the set of four state variables but differs in the assumptions on dissolution kinetics (model alternative M_lit_).

M_lit_: In literature, the dissolution term as defined in Eq. 2 and 3 is simplified to follow a first-order rate model driven by the gradient between maximal soluble PAH (*C*_*sol*_) and the dissolved PAH (*C*_*diss*_) (Perry 1950). Note that this approach does not consider the limited availability of PAH in the closed batch system but has been shown to explain dissolution kinetics in earlier studies, e.g., Volkering et al. (1992), Grimberg et al. (1996). Therefore, the dissolution term in Eqs. 2 and 3 is slightly adjusted in the differential equations for the change of the dissolved PAH and the pure PAH:

6 $$\begin{aligned} \frac{d{C}_{diss}}{dt}={k}_{diss}\bullet a\bullet \left({C}_{sol}-{C}_{diss}\right)\\-\frac{{k}_{gr}}{{Y}_{bio}} \bullet \left(B \bullet {10}^{-9}\right)-{k}_{gas}\bullet \left({C}_{diss}\bullet H-{C}_{gas}\right) \end{aligned}$$

7 $$\frac{d{C}_{crystal}}{dt}=-{k}_{diss}\bullet a\bullet \left({C}_{sol}-{C}_{diss}\right)$$

Sensitivity: Volatilization of PAHs plays a significant role under environmental conditions when the volume of the atmospheric compartment and, thus, the PAH concentration gradient from the aqueous to the gaseous phase is large (National Research Council 2003). Under laboratory batch conditions, the headspace is small compared to the atmosphere and volatilization might be an instantaneous process. The sensitivity of the model results was tested by varying the value of the mass transfer constant *k*_*gas*_ for volatilization in a range of 0.1 to 10,000 h^−1^.

### Derivation of model parameters

Data on total PAH (naphthalene) concentration (*C*_*tot*_) and microbial cells (*B*) for model calibration and parameter deviation were taken from Wang et al. (2018). Simulating a regular growth experiment where the addition of PAH marks the starting point, the state variables were initially: *C*_*crystal,0*_ = *C*_*tot,0*_, *C*_*diss,0*_ = 0, and *C*_*gas,0*_ = 0. Model alternatives (M_ref_, M_lit_) were fitted to the experimental data to derive a set of optimum model parameter values (Table S2-SI). The biomass yield (*Y*_*bio*_), the maximum growth rate constant (*q*_*max*_), and the half saturation (Monod) constant (*K*_*m*_) as well as the mass transfer constant for degassing of naphthalene into the gas phase (*k*_*gas*_) and the coefficient for dissolution of naphthalene into the aqueous phase (*k*_*diss*_*)* alongside with the specific surface area (*a)* were estimated using nonlinear least-squares fitting implemented in the computer software MATLAB (lsqnonlin). Lower (0 for all parameters) and upper boundaries (1 h^−1^ for *q*_*max*_, 1000 for all other parameters) for the parameter values were defined for the fitting procedure to limit the search space. To avoid overemphasizing the biomass data during the fitting procedure due to comparably high cell concentration versus total PAH concentration, biomass and naphthalene concentration data (both measured and simulated) were normalized by the respective maximum measured value.

For model comparison, the Akaike Information Criterion (AIC), Bayesian Information Criterion (BIC), and the normalized root-mean-square error (NRMSE) were used as indicators for the goodness-of-fit of the models to experimental data. Additionally, AIC_norm_ and BIC_norm_ were calculated using normalization by the respective maximum measured value in order to equally regard errors in all model parameters, regardless of their absolute value. All indicators consider the deviation of the model results from the actual data points. AIC is a relative indicator for the model fit and takes the number of fitted parameters and the size of the underlying dataset into account. It rewards the goodness of fit to the data while punishing model complexity, i.e., a high number of fitted parameters that might lead to overfitting. BIC, similar to AIC, uses the maximum log-likelihood to evaluate and compare different models. In contrast to AIC, however, it punishes complexity even more by giving models with an increased number of model parameters a higher score. NRMSE, AIC, and BIC are smallest for the most appropriate model. In addition to the statistical criteria, a graphical analysis, undertaken by inspecting the visual deviations between model results and measurements, supports the determination of the most appropriate model approach.

### Model application for experimental design

The optimized model was used to analyze six setups (model scenarios) for microbial laboratory-based degradation experiments that differ in PAH dosage and pulsing frequency by simulating naphthalene degradation and growth of the model organism *Cycloclasticus* sp. strain P1 (Table 1). A common experimental setup of a one-time high-dosage PAH-degradation experiment, reflecting, e.g., an accidental oil spill, was compared to scenarios where the same total mass of naphthalene was applied at equal intervals over the total experimental time of 10 days, representing e. g. repeated river runoff. For the one-time high-dosage scenario (S1), 500% of the calculated naphthalene solubility in the given system was selected (solubility = 28.96 mg L^−1^ with salinity 0.612 mol L^−1^ and Setschenow constant for naphthalene in seawater 0.256 L mol^−1^; Xie et al. 1997). Correspondingly, for the three repeated low-dosage scenarios subsequently lower concentrations per dosage were chosen. Two times 250% (S2), five times 100% (S3) and ten times 50% (S4) of 28.96 mg L^−1^ were added at equal intervals during the simulated experiments, thereby predicting biomass dynamics and naphthalene concentration in the different states (crystalline, gaseous, dissolved).

**Table 1:** Overview of the dosage-schemes in different simulated scenarios with the reference model

Scenario	Number of dosages	Dosage interval [days]	Dosage concentration [mg L^−1^]	Total simulated incubation time [days]
S1	1	-	144.79	10
S2	2	5	72.40	10
S3	5	2	28.96	10
S4	10	1	14.48	10
S5	10	1	14.48	16.9
S6	10	6.12	14.48	55.1

Degradation performance and the recovery of the batch culture after a repeated low-dosage contamination was investigated by simulating how long it would take to have the added naphthalene degraded to the same extent as in S1 after 10 days. Therefore, for scenario S5, a simulation of S4 was run with low PAH pulses equally added during the 10 days of the experiment and, afterwards, biomass and PAH dynamics were left unaffected by further inputs. Recovery time was measured while the residual naphthalene slowly faded. For scenario S6, S5 was adjusted as such that time between pulses was elongated, allowing the system to recover before a new pulse was added.

All simulation results were plotted using MATLAB. Figure 1 was created in Microsoft PowerPoint.

## Results

### Reduced complexity of the reference model

**Fig. 2:**
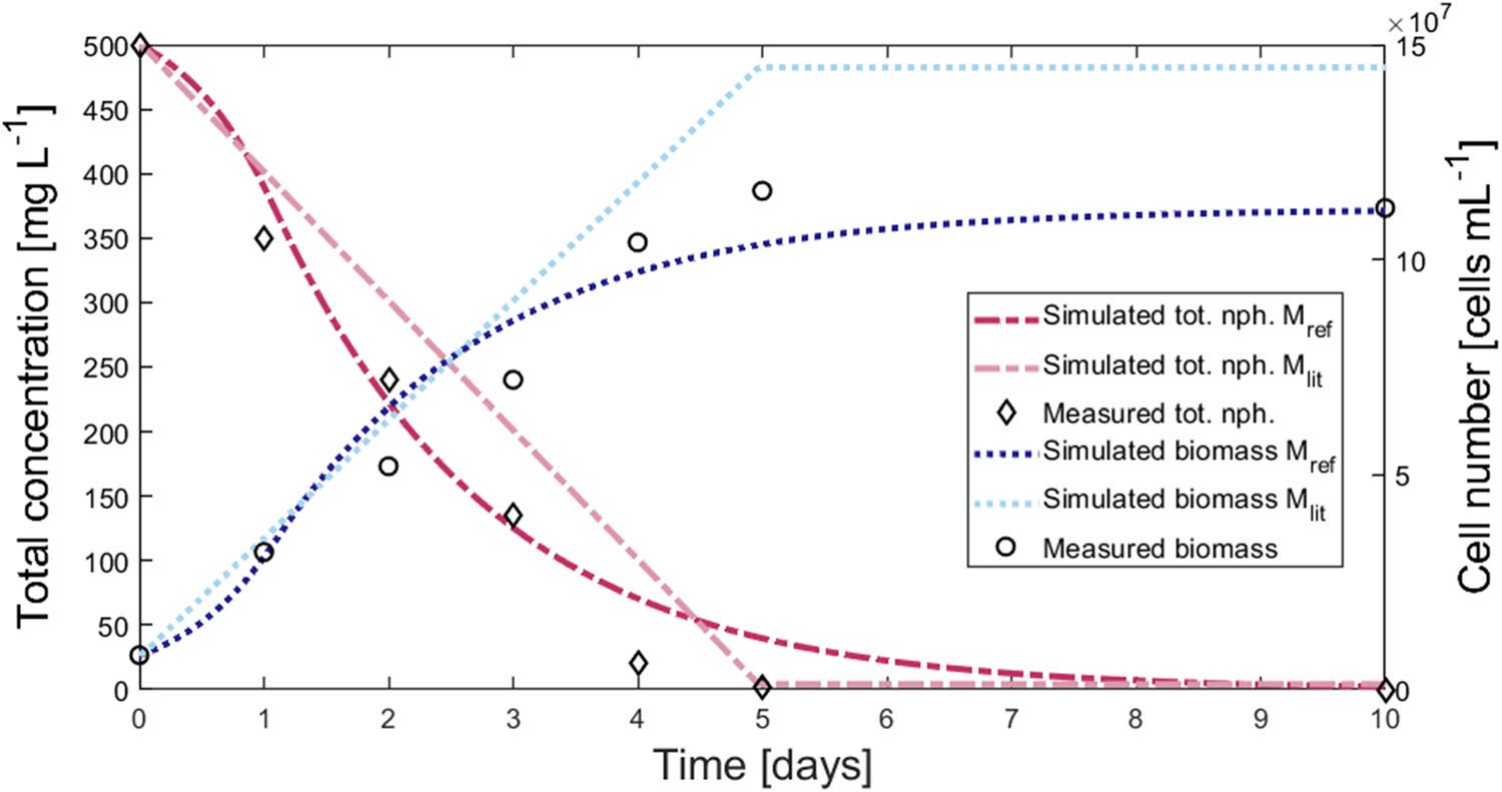
Comparison of simulated biomass (blue) and naphthalene (pink) concentrations over time by M_ref_ (dark color) and M_lit_ (lighter colour) to experimental results from Wang et al. 2018

Both model structures, i.e., M_ref_ and its alternative M_lit_, were fitted to the available experimental data from Wang et al. (2018) to simulate naphthalene degradation in a closed batch system by *Cycloclasticus* sp. strain P1. All indicators for the goodness of fit AIC/AIC_norm_, BIC/BIC_norm_, and NRMSE indicated relatively good fits and were in favor of M_ref_ (Table 2). Additionally, plotting the simulated biomass and total naphthalene concentrations of the two models in comparison to the experimental data from Wang et al. (2018) showed that M_ref_ represented the data better than M_lit_ (Fig. 2). This means that the statistical criteria fit the observations if the availability of the crystalline PAH, *C*_*crystal*_, is assumed to be the limiting factor for the dissolution kinetics and *C*_*sol*_ is the upper boundary, i.e., the capacity-term of the dissolved naphthalene concentration.

**Table 2:** Fitted parameters and goodness-of-fit indicators (*NRMSE* normalized root-mean-square error, *AIC* Akaike Information Criterion, *BIC* Bayesian Information Criterion, *AIC*_*norm*_ normalized AIC, *BIC*_*norm*_ normalized BIC) for the simplified reference model M_ref,s_ and the alternative model structure M_lit_. Specific surface area *a* and *k*_*gas*_ were set to 1 when model complexity of the reference model M_ref_ was reduced to M_ref,s_ n.a. – no literature data available

Parameter	Literature values	M_ref,s_	M_lit_	Parameter description
*q*_*max*_ [h^−1^]	0.3^a^ – 0.636^b,c^	0.281	0.543	Max. growth rate constant^a,b,c^
*K*_*m*_ [mg_NAP_ L^−1^]	0.291^c^ – 0.572^b,c^	1.000	0.499	Monod constant, half-saturation conc.^b,c^
*Y*_*bio*_ [mg_BIO_ mg^−1^_NAP_]	0.17^d^ – 0.775^c^	0.208	0.276	Biomass yield^b,c,d,e^
*k*_*diss*_ [cm h^−1^]	1.83E-3^f^	2.5E-2	1.86E-1	Mass transfer coefficient for dissolution of PAH into aqu. phase, dependent on PAH particle size and diffusion coefficient^a,f,g^
*a* [cm^−1^]	n.a	1	0.775	Specific surface area of PAH
*k*_*aq*_ = *k*_*diss*_* · a* [h^−1^]	n.a	0.025	0.144	Mass transfer constant for dissolution of PAH into aqueous phase
*k*_*gas*_ [h^−1^]	n.a	1	0.856	Mass transfer constant for degassing of PAH into gas phase
NRMSE [-]		2.725	4.446	Normalized root-mean-square error
AIC [-]		323.7	334.2	Akaike Information Criterion
BIC [-]		323.4	333.9	Bayesian Information Criterion
AIC_norm_ [-]		145.8	156.3	Akaike Information Criterion, normalized by max. values
BIC_norm_ [-]		145.6	156.0	Bayesian Information Criterion, normalized by max. values

^a^Volkering et al. (1992), ^b^Knightes et al. (2003), ^c^Knightes et al. (2000) ^d^Annweiler et al. (2000), ^e^Bouchez et al. (1996), ^f^Grimberg et al. (1996) this value was estimated for phenanthrene in a non-marine system and is given as a measure for an order of magnitude since no naphthalene specific data were available, ^g^Mulder et al. (2001); (Brimo et al. 2016)

Additionally, the model structure of M_lit_ resulted in a discontinuous development of simulated biomass and naphthalene concentrations around day 5 when crystalline naphthalene concentration was almost zero (Fig. 2 and Fig. S1-SI). Since the dissolution term of M_lit_ (Eqs. 6 and 7) is driven by the gradient between maximal soluble naphthalene (*C*_*sol*_) and the dissolved naphthalene (*C*_*diss*_), a conditional assignment was created for the case where ${C}_{crystal}>{k}_{diss}\bullet a\bullet \left({C}_{sol}-{C}_{diss}\right)$ was false. This condition leads to the immediate dissolution of the remaining crystalline naphthalene in a final dissolution step before no more dissolution was possible, since there was no crystalline naphthalene left. This if-condition created an unsteady development of the crystalline and dissolved naphthalene concentrations, which in turn affected the concentrations in gaseous concentration and biomass growth. By treating *C*_*sol*_ as a capacity-term (Eqs. 2 and 3) and thereby not requiring a conditional assignment, the model structure of M_ref_ was more robust against discontinuous developments.

In order to avoid over-parameterization, the model structure M_ref_ was tested for the necessity of the processes considered, i.e., for their sensitivity towards process parameters. The specific surface area *a* of naphthalene—a parameter which is very challenging to estimate in a laboratory experiment and for which no literature data for the given conditions could be found—was arbitrarily set to 1 with the product of *a* and *k*_*diss*_ fitted as a bulk parameter constant (*k*_*aq*_ in h^−1^). Fitting the model with both free parameters vs. keeping one parameter constant at 1 and fitting the other, resulted (and needs to result) in the same product of *k*_*diss*_* ∙ a* and improved goodness-of-fit indicators due to the reduction in parameters to be fitted and, thus, in model complexity. A sensitivity analysis for *k*_*gas*_, testing values between 0.1 and 10 000 h^−1^, showed that the model results are insensitive to changes in *k*_*gas*_ (Fig. S2-SI and Fig. S3-SI, Table S3-SI). Considering a low *k*_*gas*_, meaning a kinetic inhibition of degassing, is physico-chemically not reasonable and not observed in the laboratory. Thus, *k*_*gas*_ was set to 1 h^−1^ and thereby excluded from further simulations which resulted in the following simplified model structure M_ref,s_ of the reference model.

8 $$\frac{dB}{dt}={k}_{gr}\bullet B$$

9 $$\begin{aligned} \frac{d{C}_{diss}}{dt}={k}_{aq}\bullet {C}_{crystal}\bullet \left(1-\frac{{C}_{diss}}{{C}_{sol}}\right)\\ -\frac{{k}_{gr}}{{Y}_{bio}} \bullet \left(B \bullet {10}^{-9}\right)-\left({C}_{diss}\bullet H-{C}_{gas}\right) \end{aligned}$$

10 $$\frac{d{C}_{crystal}}{dt}=-{k}_{aq}\bullet {C}_{crystal}\bullet \left(1-\frac{{C}_{diss}}{{C}_{sol}}\right)$$

11 $$\frac{d{C}_{gas}}{dt}={C}_{diss}\bullet H-{C}_{gas}$$

12 $${k}_{gr}={ q}_{max} \bullet \left(\frac{{C}_{diss}}{{K}_{m}+{C}_{diss}}\right)$$

with the bulk dissolution rate constant *k*_*aq*_ in h^−1^ (which is, internally, a function of the specific PAH surface area *a* in cm^−1^ and the dissolution coefficient *k*_*diss*_ in cm h^−1^, ${k}_{aq}={k}_{diss}\bullet a)$.

### Derived model parameters

The resulting set of fitted model parameters for both model structures, the simplified reference model M_ref,s_ and M_lit_, was in good agreement with and in the same order of magnitude (except *k*_*diss*_) as the available literature data, i.e., for *q*_*max*_, *K*_*m*_, *Y*_*bio*_ (Table 2). The biomass independent maximum growth rate constant per biomass yield (*q*_*max*_/*Y*_*bio*_) resulted in 1.35 mg NAP (M_ref,s_) to 1.97 mg NAP (M_lit_) per mg microbial biomass and hour (literature: 0.39–3.74 mg_PAH_ mg_BIO_^−1^ h^−1^). The dissolution rate coefficient *k*_*diss*_ was overestimated by one (M_ref,s_) to two (M_lit_) orders of magnitude in comparison to the only available literature value, which was, however, determined for phenanthrene in freshwater medium (Grimberg et al. 1996). Literature values describe kinetics observed in PAH degradation studies in soils, sediment, or freshwater. This might explain the deviation, especially for dissolution kinetics for a marine water column system with high initial naphthalene concentration. Specifically, degradation studies by *Cycloclasticus* sp. strain P1, the model organism investigated in the underlying studies for this model application, are yet to be conducted.

### One-time high-dosage vs. repeated low-dosage

**Fig. 3:**
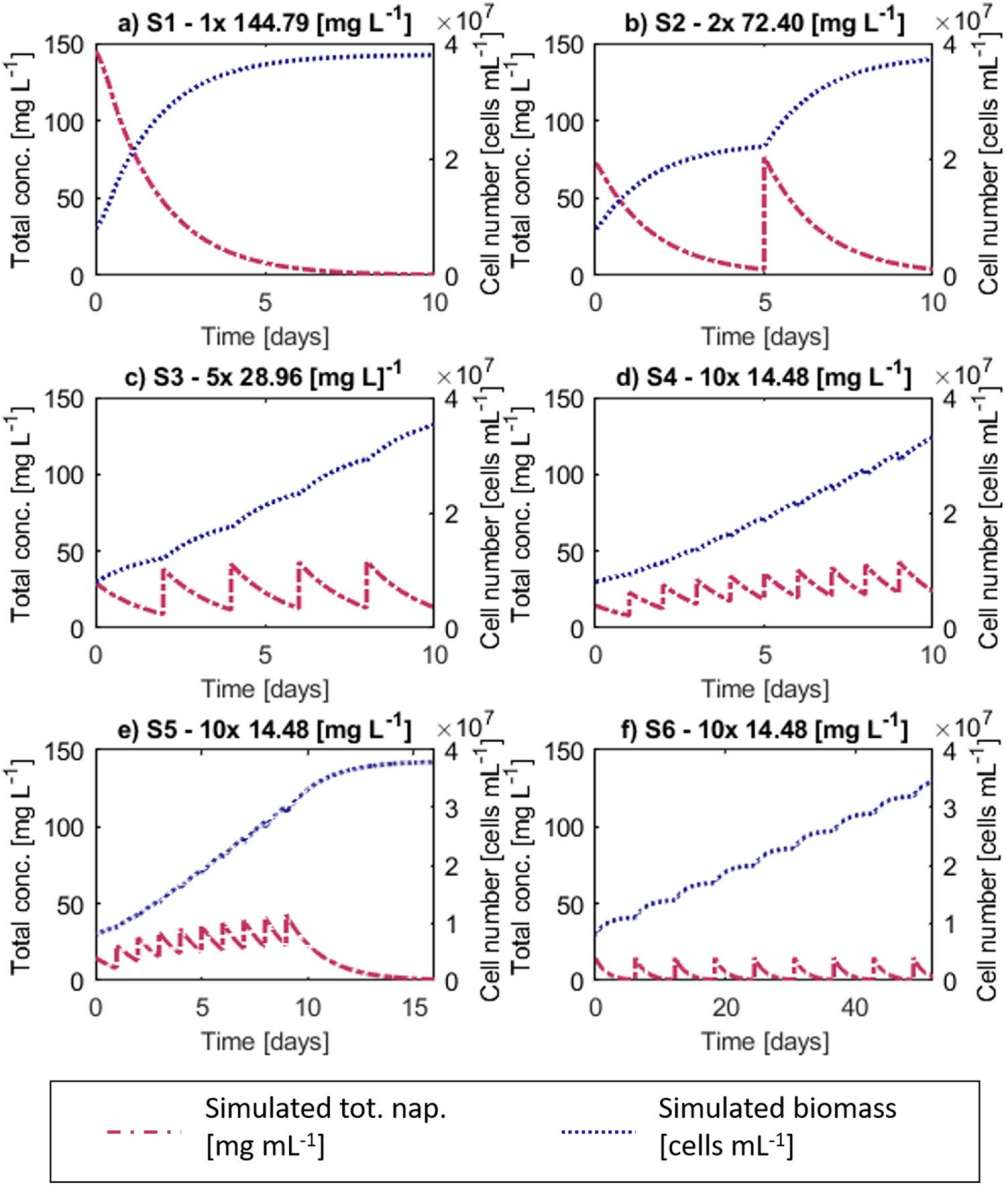
Total naphthalene (pink, dot dash) and biomass concentrations (blue, dotted) over time for the investigated scenarios. Note that x-axis for fading and elongating scenarios (S5 and S6) differ, indicating longer running times. Simulated biomass and naphthalene concentration over time for different input) scenarios. S1: High input scenario one-time dosage of 144.79 mg L^−1^ (500% max. solubility concentration). S2: Two medium dosages of 72.40 mg L^−1^ (250% max solubility concentration). S3: Five dosages of 28.96 mg L^−1^ (100% max. solubility concentration). S4: Ten dosages of 14.48 mg L^−1^ L (50% max. solubility concentration). S5: After ten dosages of 14.48 mg L^−1^ with a daily frequency, PAH degradation takes about 16 days to reach the same concentration as in S1 (0.4 mg L^−1^). S6: Low-concentration pulse (14.48 mg L^−1^) is degraded to the same PAH concentration as at day 10 in S1 (0.4 mg L.^−1^) before the next pulse is given to the system (incubation time for degradation of ten pulses 55 days)

Using the model to compare naphthalene degradation and biomass growth of *Cycloclasticus* sp. strain P1 in one-time high-dosage and repeated low-dosage batch cultures showed that a high PAH contamination scenario is potentially degraded faster than regularly pulsed low-concentration emissions due to the increased dissolution kinetics that dominate substrate availability. This is reflected in four different contamination scenarios (Fig. 3a–d). Simulating an incubation time of 10 days, highest biomass concentration (3.80·10^7^ cells mL^−1^) and lowest naphthalene residuals (99.7% degradation) were observed in, S1 where the total amount of naphthalene (144.8 mg L^−1^ = 500% maximal solubility) was added in a one-time high-dosage at the beginning of the experiment, representing a high-input event like an oil spill (Table 3). In contrast, the lowest final degradation (83.5%) was predicted after 10 days in S4, where the same total amount of naphthalene was pulsed daily (14.48 mg L^−1^ each = 50% of maximal solubility) to represent a chronic, diffuse naphthalene source such as repeating emissions. A similar final magnitude of biomass was reached at the end of all scenario simulations, while the concentration of PAH residuals varied remarkably between 0.4 mg L^−1^ (0.3% of the total added PAH amount in scenario S1) and 24 mg L^−1^ (16.5% of the total PAH amount in scenario S4; Table 3). Growth patterns and degradation dynamics were highly influenced when the total substrate mass was pulsed in partial quantities rather than being available at once in the beginning (Fig. 3a–d). The higher the frequency of the pulses the more naphthalene accumulated since microbial growth and degradation kinetics in between two pulses could not counterbalance the addition of naphthalene into the batch system. Thus, recovery of the system for chronic contamination was investigated with additional model scenarios.

**Table 3:** Comparison of simulated final naphthalene (nap) and biomass concentrations, overall nap mass loss (degradation) at the end of the simulation and incubation times for the investigated scenarios. Generation times were calculated to highlight retardation of growth despite the similar final biomass concentrations in the different scenarios

Scenario	Incubation time [days]	Final biomass concentration [cells mL^−1^]	Final nap. concentration [mg L^−1^]	Nap. degradation [%]	Generation time [days]
S1: 1 × 144.8 [mg L^−1^]	10	3.80·10^7^	0.42	99.7	0.65
S2: 2 × 72.40 [mg L^−1^]	10	3.72·10^7^	4.03	97.2	1.31
S3: 5 × 28.96 [mg L^−1^]	10	3.53·10^7^	13.4	86.6	3.17
S4: 10 × 14.48 [mg L^−1^]	10	3.31·10^7^	23.8	83.5	3.81
S5: 10 × 14.48 [mg L^−1^]	16.9	3.80·10^7^	0.42	99.7	3.81
S6: 10 × 14.48 [mg L^−1^]	55.1	3.50·10^7^	0.40	99.7	14.15

### Higher recovery time for less-frequent pulsing

The recovery of the system was investigated by adapting the repeated low-dosage PAH contamination scenario, S4. The amount of naphthalene per dosage (14.48 mg L^−1^) remained constant, but the simulations were given more time in order to reach the same extent of naphthalene degradation as observed in the one-time high-dosage scenario S1 (99.7%). In S5, where the pulsing frequency was not altered, but the system was given more time to recover after the last pulse, an additional recovery time of 6.85 days was predicted (Table 3). Moreover, when waiting for the system to fully recover each time before adding the next pulse in S6, an even greater elongation of the experimental time was predicted. The estimated time between each pulse was extended to 6.12 days, leading to a prolongation of the total simulation with ten pulses of 45.13 days in comparison to the initial 10 days of simulation. This indicates that not only naphthalene concentration, but also pulsing frequency highly influences the system recovery.

## Discussion

### Comparison of high one-time and low repeated dosages

One-time highly concentrated PAH emissions are degraded more quickly than repeatedly introduced low-concentration emissions due to the dissolution kinetics that dominate the substrate availability. The gradient between pure and dissolved PAH was the main driving force for the degradation kinetics in a closed batch system. The greater the concentration difference between the pure phase reservoir and the dissolved PAH, the faster the dissolution kinetics (according to Fick’s first law). If *C*_*crystal*_ is low (at low-contamination events or at the end of a high-level contamination event) and degradation of the dissolved PAH concentration is limited by the availability of PAH in solution, the fast degradation kinetics lead to a continuously low concentration in the dissolved, bioavailable PAH pool (*C*_*diss*_). This small gradient between *C*_*crystal*_ and *C*_*diss*_ caused dissolution and the emptying of the PAH reservoir to slow down. As a consequence, this led again to a smaller dissolved, and thereby bioavailable substrate fraction.

A low dissolved substrate concentration results in a retarded increase in biomass, since growth of the bacteria is directly dependent on the bioavailable substrate concentration (Volkering et al. 1993).

This means, that biomass growth alone is not a conclusive measure for PAH degradation in batch experiments. All the simulated scenarios, S1 to S4, had the same order of magnitude of biomass concentration at the end of the simulations. Nevertheless, 99.7% of naphthalene was degraded in S1 and the simulated bacteria, with a generation time of 0.65 days, clearly reached stationary phase in the end. In contrast, only 83.5% of the added naphthalene was degraded in S4, accumulating over time, and the simulated batch culture grew linearly, with a generation time of 3.81 days, not reaching stationary phase by the end of the simulation. This effect was described 30 years ago by Volkering et al. (1992), who found that the growth rate of PAH-degrading microorganisms and the solubility of the substrate are correlated. Their conclusion was that bioavailability is constrained due to, e.g., low solubility of the substrate which restricts microbial growth and leads to a linear increase in biomass, rather than an exponential one. In our study, the slowed growth kinetics, reflected by an increased generation time in the less contaminated scenarios, lead to retarded degradation and thereby an accumulation and higher naphthalene residuals over time. In their review, Johnsen and Karlson (2007) describe how diffuse, low-concentration contamination of PAHs can become enriched in pristine environments like soils despite the presence of PAH-degrading organisms, due to limited bioavailability of the substrate, in this case caused by sorption. Indeed, breaking the established gradient of a system by mixing is a well-known measure for increasing biodegradation. For example, the constant shaking of bacterial cultures in laboratory experiments or mixing contaminated soil with air in remediation scenarios are processes that enhance the bioavailability of the electron donor or acceptor (PAH and/or oxygen), promoting biodegradation (Sales da Silva et al. 2020). This highlights the importance of both, (1) studying pollutant degradation over longer time spans, even under controlled batch conditions, and (2) measuring hydrocarbon concentration rather than using abundance of PAH-degrading organisms as an indicator for degradation kinetics. So far, this has been discussed but rarely applied in microbial field studies, due to the challenges connected to *in-situ* hydrocarbon degradation experiments (Gontikaki et al. 2018; Liang et al. 2019). However, as shown with the simulation studies here, only observing biomass abundance could lead to an overestimation of naphthalene degradation in low-contamination scenarios.

Depending on the pulsing frequency, low-concentration hydrocarbon pulses could lead to a long-term accumulation of the pollutant rather than nearly complete degradation as described for several pristine areas like alpine lakes or mountain areas (Carrera et al. 2001; Vilanova et al. 2001).

System recovery takes longer for repeated low-concentration contamination events. Lower pulsed PAH concentration lead to less dissolved substrate that is available for PAH-degrading bacteria. This, in turn, prevents PAH-degrading bacteria from growing to high densities during the short times which is a self-enforcing process, and ultimately leads to a retardation of the system’s recovery. This effect is amplified when increasing the time between dosages (comparing, e.g., the generation times and incubation times of S5 and S6, Fig. 3e and f, Table 3). The implication is that for a pristine marine system, less frequent low-input contamination takes longer to fully recover from or might even lead to pollutant accumulation in the long-run because the PAH-degrading community is not well established due to low growth rates, inhibited by low substrate availability (Carrera et al. 2001; Vilanova et al. 2001). Given that most anthropogenic PAH contaminations in marine environments occur at low concentration and on a relatively continuous, repeated basis, the environmental relevance for PAH degradation dynamics is significant.

### Limitations of the model

Since the model itself as well as the underlying degradation experiment are simplifications of the environmental conditions and processes, the chosen model structure was based on a set of boundary conditions and, therefore, comes with limitations. Model outcomes in this study represent homogeneous, laboratory batch experiments. Since, in this study, the general degradation dynamics and growth patterns were of importance, not the absolute concentration values, only the dissolved fraction of the PAH is considered to be bioavailable without restriction of the general validity. Nevertheless, under environmental conditions, additional processes like sorption to solid particles, to dissolved organic matter or to biomass like dead cell walls or EPS in bioaggregates might play a role and could even increase the effects we observed, since bioavailability of the PAH would be even lower (Volkering et al. 1992). Microbial degradation of PAHs might be adversely affected by the PAH concentration itself, e.g., by a toxicity effect at high PAH concentration or by a concentration threshold below which bacteria stop degrading the PAHs, though no such effects have been reported for naphthalene in the literature so far. In addition, dissolution can be actively enhanced by bacteria, e.g., due to the excretion of biosurfactants and bioemulsifiers (Bozzi et al. 1996; Grimberg et al. 1996; Mulder et al. 1998; Qin et al. 2007). However, Gutierrez et al. (2018) found that *Cycloclasticus* spp., isolated near the highly damaged Deepwater Horizon oil rig in 2010, were not capable of producing such biosurfactants, so this was neglected for the PAH biodegradation by *Cycloclasticus* spp. in our model. When extending the model to include several bacterial strains or a complex microbial community, the effect of biosurfactants should be considered since the same study suggested that *Cycloclasticus* spp. jointly with other oil-degrading bacteria were able to benefit from the emulsifying properties of such biosurfactants.

Different physico-chemical properties of aromatic pollutants other than naphthalene could change the observed degradation behavior. Given that naphthalene is a relatively hydrophilic PAH, resulting in a comparably high solubility (even in seawater), the one-time high-dosage scenario that showed a fast degradation process might also be retarded for substances with slower dissolution kinetics (Volkering et al. 1992). Nevertheless, the relative pattern between the input scenarios theoretically should not change when transferring the results for naphthalene to other organic compounds if the conditions are kept the same. Mulder et al. (2001) simulated the biodegradation of four PAHs with different mass transfer parameters such as water solubility and sorption coefficients and found similar patterns of degradation, which were governed by the physico-chemical properties of the PAHs. They concluded that extrapolating kinetic information for different PAHs can be done under mass-transfer limited conditions because the mass-transfer processes are then dominated by the physico-chemical properties of the pollutants.

The elongated degradation times and accumulation of PAHs that we observed for repeated low-concentrated hydrocarbon input is especially relevant for pristine environments. These rely on the establishment of a PAH-degrading community for decontamination, which in turn needs PAH to grow and sustain itself. Studies suggest, that in chronically polluted areas where low levels of PAHs constantly exist, the native bacterial community adapts and enriches PAH-degrading organisms, probably from the “rare biosphere” and is therefore able to respond faster to PAH contamination (King et al. 2015; Kleindienst et al. 2016; Cerro-Gálvez et al. 2021). Thus, the observed effects from our study are potentially not as severe for such chronically polluted environments and further simulations should investigate this aspect.

In marine environments, contamination is rarely caused by a single PAH, but a complex hydrocarbon mixture like crude oil or diesel, containing aliphatic, branched, cyclic, and polyaromatic hydrocarbons which interact and influence the mass transfer between the compartments of the system (Ghoshal and Luthy 1998; Guha et al. 1999; Ribicic et al. 2018). On top of this, not a single strain, but a complex community evolves to degrade the compounds, all influenced by environmental factors like salinity, UV light, pressure, shoreline energy, mineral particles, nutrients, and temperature (Bargiela et al. 2015; Ward et al. 2018; Piccardi et al. 2019; Bacosa et al. 2022). To begin to understand the effect of (re-occurring) low-concentration oil contamination and expand the model to account for all these variables, further research both in the field and in the laboratory is necessary to tease apart the tangled interlinkages between them.

## Conclusion

The model outcomes in this study indicate that it takes longer for a system to recover from less frequent and repeated low-concentration hydrocarbon contamination events. Therefore, events like contamination due to shipping might lead to pollutant accumulation over time. These findings are even more relevant, considering that low-concentration hydrocarbon contamination events occur more often than one-time high-dosage contamination events like accidental oil spills. Additionally, such diffuse events are, in general, a challenge to monitor and, thus, are overlooked. Furthermore, pristine environments are more likely to suffer from low-concentration hydrocarbon contamination since the system is not adapted to PAH-input and, therefore, PAH-degrading bacteria are potentially less abundant and active. This highlights the vulnerability of regions like the Arctic Ocean towards even sporadic low-dosage hydrocarbon contamination. Ultimately, awareness needs to be raised and efforts need to be increased to protect such vulnerable areas against diffuse chronic contamination.

Further studies are needed to investigate contaminated marine systems with high temporal and spatial resolution. More complex experiments and models with PAH mixes or crude oil as well as field studies in marine habitats are required to discern the bioavailability and degradation efficiency of low-concentrated hydrocarbon contamination. This is of particular importance considering the different parameters that influence the bioavailability of hydrocarbons such as the solubility of PAHs in the presence of co-solvents, the salt concentration, and the presence of sediment particles or biosurfactants. Field campaigns that sample habitats influenced by regular low-concentration hydrocarbon contamination—e.g., shipping routes—over timescales from days to months could determine disruptions and in situ recovery times for these ecosystems that might also adapt to the pollutant pressure. Collectively, these data would be needed to confirm the trends we simulated and to increase the model complexity, i.e., to test our advances in system understanding. Our study is the foundation that can be used to better constrain the interactions of coupled pollutant degradation and microbial growth processes and may have important implications for future management and risk assessment of diffuse oil contamination in the marine environment.

## Supplementary Information

Below is the link to the electronic supplementary material.

Supplementary file1 (PDF 615 KB)

## Data Availability

The MATLAB code used to generate the simulations in this study is available from the corresponding author on reasonable request.
