# Supplementary material for: Dosage concentration and pulsing frequency affect the degradation efficiency in simulated bacterial polycyclic aromatic hydrocarbon-degrading cultures
Source: Environ Sci Pollut Res Int. 2023 Apr 4;30(21):59813–25. doi: 10.1007/s11356-023-26546-9 (PMC10163121; doi:10.1007/s11356-023-26546-9)
Supplement: Supplementary file 1 — Supplementary file1 (PDF 615 KB) [file 11356_2023_26546_MOESM1_ESM.pdf]

Supplementary Information

Dosage concentration and pulsing frequency affect the degradation efficiency in simulated bacterial polycyclic aromatic hydrocarbon-degrading cultures

Anjela L. Vogel[1], Katharine J. Thompson[1], Sara Kleindienst[1,2], & Christiane Zarfl[1]

[1]Eberhard Karls University of Tübingen, Department of Geosciences, Schnarrenbergstr. 94-96, 72076 Tübingen, Germany

[2]Department of Environmental Microbiology, Institute for Sanitary Engineering, Water Quality and Solid Waste Management (ISWA), University of Stuttgart, Germany

Environmental Science and Pollution Research

Corresponding author:

Anjela L. Vogel

anjela.thon@uni-tuebingen.de, phone: +40 7071 29 74708

Dosage concentration and pulsing frequency affect the degradation efficiency in simulated bacterial polycyclic aromatic hydrocarbon-degrading cultures - SI texts

$$\frac{cells}{mL} = OD * 8 * 10^8 \tag{ES1}$$

Conversing OD values into cell numbers can be done with a linear equation, when more cells correspond to higher turbidity, which is the case for many cultures growing on aqueous medium during exponential phase (Madigan et al., 2010).This conversion is depending on factors like cell size and shape, the used growth medium and substrate and therefore should be calculated for every investigated system by conducting a calibration experiment. But since those data are not available for the experimental data from Wang et al. (2018), we resorted to using a linear relationship from a lab protocol, which is commonly used for *E.coli* to convert cell densities obtained by measuring the optical density photo-spectrometrically in our lab (Equ. ES2). This general conversion was applied, assuming the resulting cell numbers are at least in the right order of magnitude and nonetheless illustrate the trend the biomass concentration follows over time. Comparing the resulting cell numbers (Table S1) to our own growth experiments with *Cycloclasticus pugetii* PS-1, a very close relative of *Cycloclasticus* sp. P1 (100% nucleotide pairwise identity of the 16S rRNA genes, 88% nucleotide pairwise identity of the available genomes due to NCBI data base August 2022), the calculated cell numbers were reasonable (Vogel et al., in prep.).

Equation to estimate maximal solubility concentration in seawater c_{sol}^{sea} (mg L^{-1}) based on the maximal solubility concentration in freshwater c_{sol}^{fresh} (mg L^{-1}), the salt concentration in the seawater C_{sal} (mol L^{-1}), and the Setschenowv constant k_s (L mol^{-1}) (Gold et al., 1989; Xie et al., 1997).

$$c_{sol}^{sea} = c_{sol}^{fresh} * e^{\left(\frac{(-k_s * C_{sal})}{2.3}\right)} \tag{ES2}$$

Dosage concentration and pulsing frequency affect the degradation efficiency in simulated bacterial polycyclic aromatic hydrocarbon-degrading cultures - SI Figures

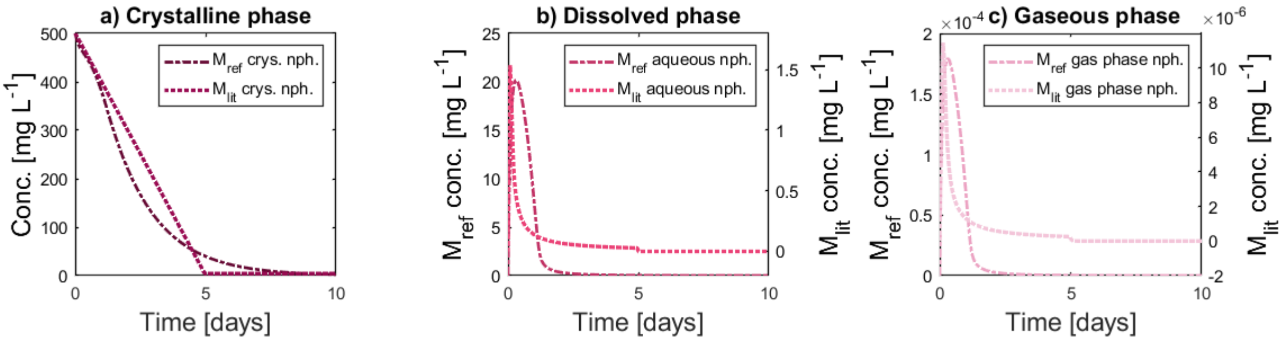

Fig. S1 Comparison between M_{lit} and M_{ref} of the naphthalene distribution between the crystalline, dissolved and gaseous phases in the simulated system

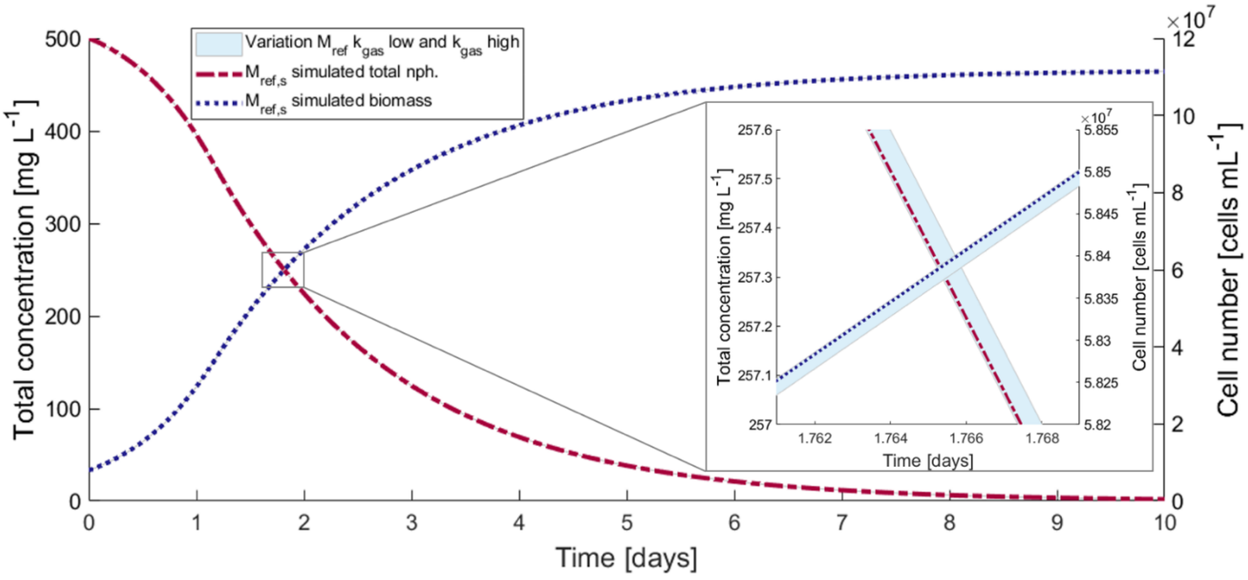

Fig. S2 Variation of $M_{ref,s}$ simulated total naphthalene and biomass concentration over time with k_{gas} low = 0.1 [1/h] and k_{gas} high = 10 000 [1/h]. Neither increasing nor decreasing k_{gas} over several magnitudes does affect the results severely

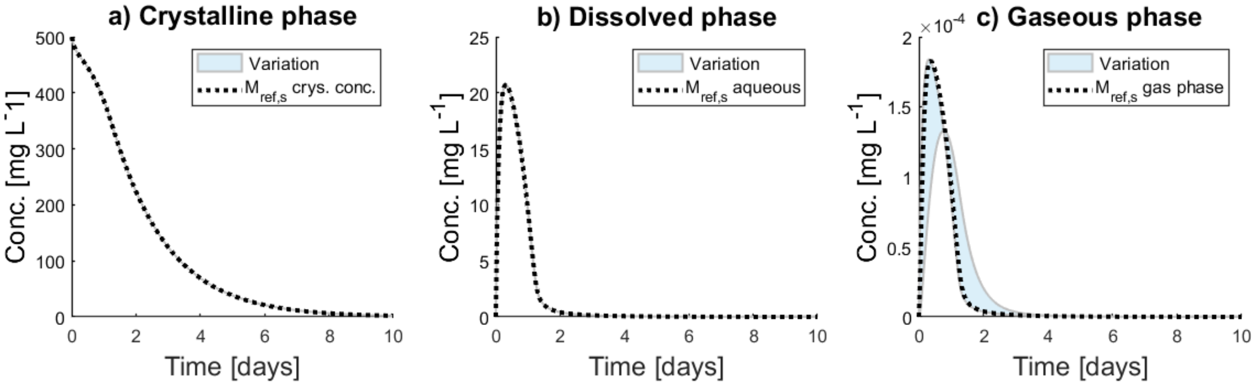

Fig. S3 Variation of M_{ref} simulated total naphthalene concentration in crystalline, aqueous and gaseous phases over time with k_{gas} low = 0.1 [1/h] and k_{gas} high = 10 000 [1/h]. Increasing k_{gas} further over several magnitudes does not affect the results visibly. Decreasing it below 1 does start to impact the distribution in the gaseous phase

Dosage concentration and pulsing frequency affect the degradation efficiency in simulated bacterial polycyclic aromatic hydrocarbon-degrading cultures - SI Tables

Table S1 Experimental results used for parameter fitting (Wang et al., 2018). Naphthalene residuals were converted to concentrations using the reported initial concentration of 500 [mg/L]. Cell densities were used to roughly estimate cell numbers in [cells mL^{-1}] using equation ES1

Days	0	1	2	3	4	5	10
Nph. residuales [%]	0	30	52	73	96	98	100
Cal. nph. conc. [mg L^{-1}][a]	500	350	240	135	20	2	0
Cell density (OD_{600})	0.1	0.4	0.65	0.9	1.3	1.45	1.4
Cell nmb. [cells mL^{-1}][a,b]	$8.0*10^7$	$3.2*10^8$	$5.2*10^8$	$7.2*10^8$	$1.04*10^9$	$1.16*10^9$	$1.12*10^9$

[a]data used for parameter fitting, [b]estimated cell numbers calculated using ES1

Table S2 Input model parameters from literature and initial values for the fitted parameters

Parameter	Value	Reference
Setschenow constant k_s for nph. in seawater [L mol^{-1}]	0.256	(Xie et al., 1997)
Max. saturation concentration c_{sol}^{fresh} in freshwater [mg_{NAP} L^{-1}]	3.10E+1	(Yalkowsky et al., 1983)
Salt concentration C_{sal} of seawater [mol L^{-1}]	0.612	(Gold et al., 1989; Xie et al., 1997)[a]
Max. saturation concentration C_{sol} of nph. (solubility in seawater) [mg_{NAP} L^{-1}]	28.96	(Gold et al., 1989; Xie et al., 1997)[b]
Henry constant H for equilibrium of nph. between water and gas phase [-]	8.88E-06	(Ma et al., 2010; Sander, 2015)
Mass of one bacterium [mg cell^{-1}]	1.00E-09	(Davis et al., 1973)
Initial conditions for parameter fitting		
Max. growth rate constant q_{max} [mg_{NAP} mg_{BIO}^{-1} h^{-1}]	0.346	(Knightes et al., 2003)
Monod half-saturation constant K_m [mg_{NAP} L^{-1}]	0.572	(Knightes et al., 2003)
Biomass yield Y_{bio} [mg_{BIO} mg_{NAP}^{-1}]	0.35	(Bouchez et al., 1996; Knightes et al., 2003)
Mass transfer coefficient for dissolution into aqueous phase k_{diss} [cm h^{-1}]	0.25	x
Specific surface area of nph. a in [cm^{-1}]	1	x
Particle independent mass transfer into aqueous phase $k_{aq} = k_{diss} \cdot a$ in [h^{-1}]	0.25	x
Mass transfer constant for degassing k_{gas} in [h^{-1}]	1	x

[a]assuming only NaCl contributes to the molarity of 3.5% salinity, [b]calculated with ES2

Table S3 Model parameter and errors of investigated model structures. Main model is $M_{ref,s}$, highlighted in light blue. Parameter values labeled with * were fixed to 1 for the respective fit in order to reduce complexity of the model structure. n.a. – no literature data available

Parameter	Literature	M_{lit}	$M_{ref,s}$	$M_{ref,2}$	$M_{ref,3}$	M_{ref}
q_{max} [mg_{NAP} mg_{Bio}^{-1} h^{-1}]	0.346 – 0.636	0.543	0.281	0.286	0.277	0.290
K_m [mg_{NAP} L^{-1}]	0.291 – 0.572	0.499	1.000	0.950	0.996	0.919
Y_{bio} [mg_{BIO} mg_{NAP}^{-1}]	0.2 – 1.3	0.276	0.208	0.208	0.208	0.208
k_{diss} [cm h^{-1}]	0.00183	0.186	0.025	0.077	0.025	0.069
a [cm^{-1}]	n.a.	0.775	1*	0.318	1*	0.347
$k_{aq} = k_{diss} \cdot a$ [h^{-1}]	n.a.	0.144	0.025	0.024	0.025	0.024
k_{gas} [h^{-1}]	n.a.	0.856	1*	1*	0.540	989.3
NRMSE [-]		4.446	2.725	2.721	2.728	2.720
AIC [-]		334.2	323.7	325.7	325.6	327.8
BIC [-]		333.9	323.4	325.5	325.3	327.5
AIC_{norm} [-]		156.3	145.8	147.9	147.8	150.0
BIC_{norm} [-]		156.0	145.6	147.6	147.5	149.6

References

Bouchez M, Blanchet D, Vandecasteele J-P (1996) The microbiological fate of polycyclic aromatic hydrocarbons: carbon and oxygen balances for bacterial degradation of model compounds. Appl Microbiol Biotechnol 45(4):556-561. https://doi.org/10.1007/BF00578471

Davis BD, Renato D, N. EH, S. GH (1973) Bacterial Physiology: Microbiology. Maryland

Gold G, Rodriguez S (1989) The effect of temperature and salinity on the Setschenow parameters of naphthalene in seawater. Can J Chem 67(5):822-826. https://doi.org/10.1139/v89-127

Knightes CD, Peters CA (2003) Aqueous phase biodegradation kinetics of 10 PAH compounds. Environ Eng Sci 20(3):207-218. https://doi.org/10.1089/109287503321671410

Ma Y-G, Lei YD, Xiao H, Wania F, Wang W-H (2010) Critical review and recommended values for the physical-chemical property data of 15 polycyclic aromatic hydrocarbons at 25 C. J Chem Eng Data 55(2):819-825. https://doi.org/10.1021/je900477x

Madigan MT, Clark DP, Stahl D, Martinko JM (2010) Brock Biology of Microorganisms, 13th ed.,. San Francisco, Benjamin Cummings

Sander R (2015) Compilation of Henry's law constants (version 4.0) for water as solvent. Atmos Chem Phys 15(8):4399-4981. https://doi.org/10.5194/acp-15-4399-2015

Vogel AL, Thompson KJ, App CB, Gutierrez T, Kleindienst S (in prep.) Constitutive expression of PAH-degradation genes by a key oil-degrader indicates limitations in their use as biomarkers for petrochemical pollution.

Wang W, Wang L, Shao Z (2018) Polycyclic aromatic hydrocarbon (PAH) degradation pathways of the obligate marine PAH degrader *Cycloclasticus* sp. strain P1. Appl Environ Microbiol 84(21):e01261-01218. https://doi.org/10.1128/AEM.01261-18

Xie W-H, Shiu W-Y, Mackay D (1997) A review of the effect of salts on the solubility of organic compounds in seawater. Mar Environ Res 44(4):429-444. https://doi.org/10.1016/S0141-1136(97)00017-2

Yalkowsky SH, Valvani S, Mackay D (1983). Estimation of the aqueous solubility of some aromatic compounds. Residue Reviews, Springer: 43-55
